# Fecal Carriage of Multidrug-Resistant Organisms Increases the Risk of Hepatic Encephalopathy in Cirrhotic Patients: Insights from Gut Microbiota and Metabolite Features

**DOI:** 10.21203/rs.3.rs-4328129/v1

**Published:** 2024-05-07

**Authors:** Peishan Wu, Pei-Chang Lee, Tien-En Chang, Yun-Cheng Hsieh, Jen-Jie Chiou, Chao-Hsiung Lin, Yi-Long Huang, Yi-Tsung Lin, Teh-Ia Huo, Bernd Schnabl, Kuei-Chuan Lee, Ming-Chih Hou

**Affiliations:** Taipei Veterans General Hospital; Taipei Veterans General Hospital; Taipei Veterans General Hospital; Taipei Veterans General Hospital; EudaiBiome; National Yang Ming Chiao Tung University - Yangming Campus; National Yang Ming Chiao Tung University - Yangming Campus; Taipei Veterans General Hospital; Taipei Veterans General Hospital; University of California San Diego; Taipei Veterans General Hospital; Taipei Veterans General Hospital

**Keywords:** Multidrug resistant organisms, cirrhosis, complications, microbiota, metabolite, hepatic encephalopathy

## Abstract

**Background:**

Impact of fecal colonization by multidrug-resistant organisms (MDROs) on changes in gut microbiota and associated metabolites, as well as its role in cirrhosis-associated outcomes, has not been thoroughly investigated.

**Methods:**

Eighty-eight cirrhotic patients and 22 healthy volunteers were prospectively enrolled with analysis conducted on plasma metabolites, fecal MDROs, and microbiota. Patients were followed for a minimum of one year. Predictive factors for cirrhosis-associated outcomes were identified using Cox proportional hazards regression models, and risk factors for fecal MDRO carriage were assessed using logistic regression model. Correlations between microbiota and metabolic profiles were evaluated through Spearman’s rank test.

**Results:**

Twenty-nine (33%) cirrhotic patients exhibited MDRO carriage, with a notably higher rate of hepatic encephalopathy (HE) in MDRO carriers (20.7% vs. 3.2%, *p* = 0.008). Cox regression analysis identified higher serum lipopolysaccharide levels and fecal MDRO carriage as predictors for HE development. Logistic regression analysis showed that MDRO carriage is an independent risk factor for developing HE. Microbiota analysis showed a significant dissimilarity of fecal microbiota between cirrhotic patients with and without MDRO carriage (*p* = 0.033). Thirty-two metabolites exhibiting significantly different expression levels among healthy controls, cirrhotic patients with and without MDRO carriage were identified. Six of the metabolites showed correlation with specific bacterial taxa expression in MDRO carriers, with isoaustin showing significantly higher levels in MDRO carriers experiencing HE compared to those who did not.

**Conclusion:**

Fecal MDRO carriage is associated with altered gut microbiota, metabolite modulation, and an elevated risk of HE occurrence within a year.

## Introduction

Cirrhosis is susceptible to infections [1]. The widespread use of antibiotics has rapidly increased the global prevalence of infections caused by multidrug-resistant organisms (MDROs) in patients with cirrhosis [2–4]. Moreover, the colonization of MDROs in such patients emerges as a critical factor for subsequent MDRO infections, contributing to a negative impact on overall survival [5–8]. The intestine serves as a main reservoir for MDROs [9], and high rates of asymptomatic intestinal carriage of MDROs have been documented in patients with cirrhosis, particularly among those awaiting liver transplantation, or experiencing hepatic decompensation or critical illness [6, 7, 10].

In non-cirrhotic hospitalized patients, the composition of intestinal bacteria differs between those with and without rectal MDRO carriage, showing decreased diversity in MDRO carriers [11–13]. Certain commensals may protect against resistant bacteria in the intestine [14, 15]. The successful decolonization of resistant pathogens in the gut through fecal microbiota transplantation further supports the role of the gut microbiota [16].

The composition of the gut microbiota in patients with cirrhosis differs from that of the general population [17, 18]. This microbiota alteration, coupled with the impaired gut barrier function found in cirrhosis, further contributes to cirrhosis-associated complications, such as spontaneous bacterial peritonitis (SBP), hepatic encephalopathy (HE), hepatorenal syndrome, and acute on chronic liver failure [18–20]. Some data have shown poor survival in cirrhotic patients colonized or infected with MDROs; however, the impact of long-term fecal colonization by MDROs on specific outcomes related to cirrhosis remains unclear. Therefore, this study aimed to investigate the roles of fecal MDRO colonization and the associated metabolites in influencing clinical outcomes associated with cirrhosis.

## Materials and Methods

### Participants and Data Collection

This prospective study was conducted at Taipei Veterans General Hospital between October 2018 and April 2022. Liver cirrhosis was diagnosed based on histological, clinical, biochemical, endoscopic, and imaging findings suggestive of cirrhosis in patients with chronic liver disease [21]. Patients with previous history of HE, active hepatocellular carcinoma status; malignancies other than hepatocellular carcinoma; with human immunodeficiency virus infection or severe comorbidities, such as chronic renal failure, heart failure, or chronic obstructive pulmonary disease; and those who received proton pump inhibitor, nonsteroidal anti-inflammatory drugs, antibiotics, or probiotics within 1 month were excluded. Twenty-two healthy adults without underlying systemic disease were enrolled as healthy controls. This study was approved by the Institutional Review Board of Taipei Veterans General Hospital (IRB No., 2017–09-013C and 2019–08-013A). Written informed consent was obtained from each participant.

Demographic characteristics, laboratory data, and medical history were collected. Blood and stool samples were collected on the day of enrollment. Stool samples from 18 hospitalized cirrhotic patients were collected in the ward, while samples from the remaining 70 cirrhotic outpatients and 22 healthy subjects were collected at home. Cirrhotic patients were followed for at least 1 year, or until death or liver transplant. During the follow-up period, clinical events were recorded, which included (1) complications of cirrhosis, such as SBP, overt HE [22], newly developed or worsening ascites, acute kidney injury, first or recurrent variceal bleeding; (2) newly diagnosed hepatocellular carcinoma; (3) bacterial infections; (4) death; or (5) liver transplant.

### Definition of MDROs

The stool samples collected were inoculated onto selective agar plates at 37°C for 24 hours to detect MDROs. MDROs were defined as microorganisms that are resistant to at least one agent in three or more antimicrobial categories as characterized earlier [23]. MDROs include methicillin-resistant *Staphylococcus aureus*, vancomycin-resistant enterococci, and multidrug-resistant gram-negative bacilli, including *Enterobacterales, Pseudomonas aeruginosa*, and *Acinetobacter baumannii*. Details of MDRO detection, processing and analysis of stool bacterial genomic data, as well as metabolite analysis are provided in Supplementary Methods.

### Measurement of lipopolysaccharides

Plasma lipopolysaccharides (LPS) were measured by enzyme-linked immunosorbent assay kits (Cloud-Clone Corp, Katy, TX, USA) according to the manufacturer’s instructions.

### Statistical Analysis

Clinical data were expressed as median (25th – 75th percentiles) or as counts, as appropriate. The chi-square or Fisher’s exact test was used to analyze categorical variables and the Mann–Whitney *U*-test was applied to assess continuous variables between MDRO carriers and non-carriers in the cirrhotic population. Cox regression analysis was performed to identify potential predictors for clinical outcomes. A logistic regression model was used to identify the risk factors for fecal carriage of MDROs. The cutoff values of Model for End-Stage Liver Disease score (MELD) and lipopolysaccharides (LPS) levels are determined using Youden’s index. Variables with *p* < 0.1 in the univariate analysis were included in the multivariable analysis. Statistical significance was defined as *p* < 0.05. The Kruskal–Wallis test was used to assess the metabolite differences among healthy controls, cirrhotic patients with and without MDROs, and among healthy controls, cirrhotic patients with and without HE. Data were considered significant when *p* < 0.05. All statistical analysis were performed using IBM SPSS Statistics for Windows, Version 24.0 (IBM Corp. Armonk, NY, USA).

Detailed analyses for the gut microbiota and the metabolites are provided in Supplementary Methods.

## Results

### Fecal Microbiological Findings in Healthy Controls and Cirrhotic patients

Eighty-eight patients with cirrhosis and twenty-two healthy volunteers were enrolled. The median age and gender distribution were similar between the two populations (**Suppl. Table 1**). Table 1 showed that the fecal MDRO colonization rate was higher in patients with cirrhosis than the healthy controls (33% vs. 9.1%, *p* = 0.026) Of the 29 cirrhotic patients with fecal MDRO carriage, 6 patients (30.7%) were colonized with ≥ 2 MDROs. The most commonly isolated MDRO was extended-spectrum beta-lactamase producing *Escherichia coli* (58.6%), followed by vancomycin-resistant *Enterococcus* spp. (48.3%).

### Characteristics and Outcomes Between Cirrhotic Patients With and Without MDRO Carriage

The demographic data of the cirrhotic patients with and without fecal carriage of MDROs are summarized in Table 2. No differences in terms of age, gender, etiology of cirrhosis, underlying comorbidities, and severity of liver disease were observed between MDRO carriers and non-carriers. MDRO carriers had higher plasma LPS levels (15.1 vs 10.4 ng/L, *p* = 0.006) and a higher proportion of admission within 30 days (34.5% vs 13.6%, *p* = 0.002) compared to non-carriers. During a median of 16.4 months follow-up (range, 0.7–40.4 months), 36 patients (40.1%) developed cirrhosis-associated complications within 1 year after enrollment (Table 3). MDRO carriers had higher rates of HE occurrence than non-carriers (20.7 vs 3.4%, *p* = 0.008). However, other cirrhotic complications, including infectious events, did not show a significant difference between MDRO carriers and non-carriers. Regarding the infectious events, 5 patients experienced SBP, while ten encountered other types of infections. Among the 10 patients with non-SBP infections, five had bacteremia, two had intra-abdominal infections, two had aspiration pneumonia, and one had a urinary tract infection. The positive culture rate for these infectious events was 53%, but none of the bacterial cultures identified were MDROs.

To identify potential predictors associated with HE within 1 year, Cox regression analysis was performed (Table 4). Univariate analysis showed that higher serum LPS levels (≥ 14.9 ng/mL), Child-Pugh class C, and fecal MDRO carriage were independent predictors for HE occurrence. Importantly, LPS ≥ 14.9 ng/mL and fecal MDRO carriage maintained their statistical significance on multivariable analysis, further supporting their association with HE occurrence. Table 5 shows the risk factors associated with fecal colonization of MDROs in cirrhotic patients. In the univariate analysis, higher serum LPS levels (≥ 11.9 ng/mL) and prior admission in the last 30 days were significant risk factors for fecal MDRO colonization. However, on multivariable analysis, prior admission within 30 days did not predict MDRO colonization. Only serum levels of LPS ≥ 11.9 ng/mL (OR = 3.84; *p* = 0.009) remained an independent risk factor for MDRO colonization.

### Fecal Microbiome Comparisons Among Healthy Adults, and Cirrhotic Patients With and Without Fecal MDRO Carriage

**Figure 1:**
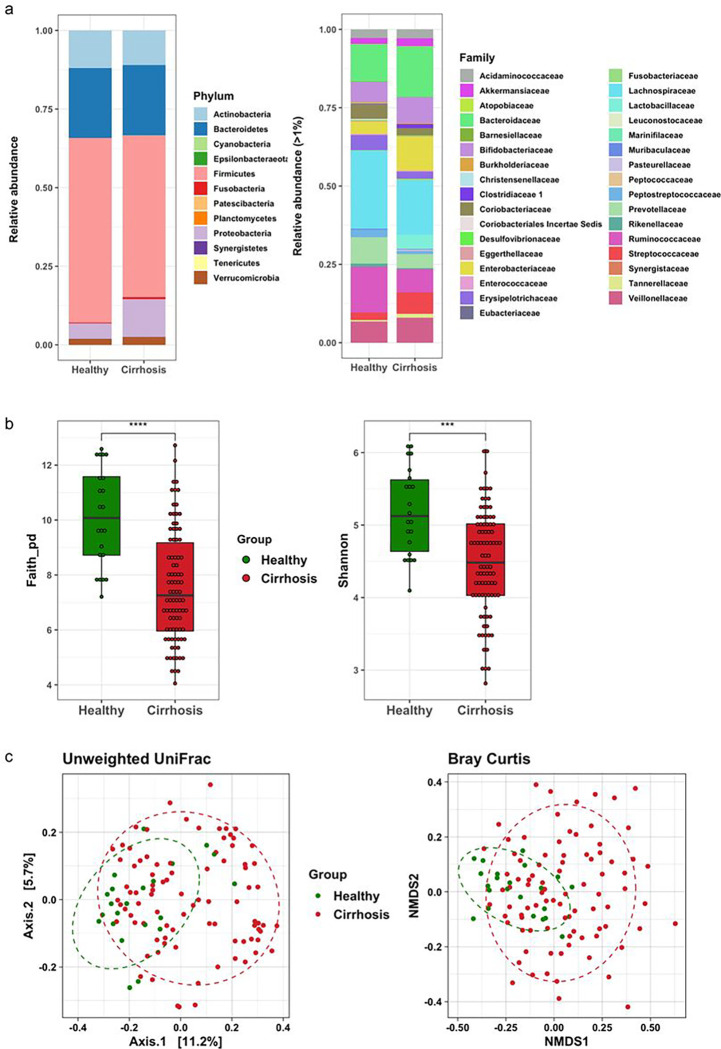
The composition and diversity of fecal microbiota in healthy subjects and cirrhotic patients. Stacked bar plots of phylogenetic composition of common bacterial taxa (> 0.1% abundance) at the (A) phylum level and (B) family level in fecal samples of normal subjects and cirrhotic patients by 16 S rRNA sequencing. **(C)** Alpha diversity indices of fecal bacteria measured by Faith’s PD index and Shannon index. (D) Principal coordinate analysis of fecal microbiota by unweighted Unifrac distance metrics and Bray–Curtis distance. **** *p* <0.0001, *** *p* <0.001

Compared with healthy adults, Proteobacteria was predominant in the fecal samples of patients with liver cirrhosis (Fig. 1a). At the family level, Bacteroidaceae, Enterobacteriaceae, Lactobacillaceae and Streptococcaceae were increased, while Lachnospiraceae and Ruminococcaceae were decreased in the feces of cirrhotic patients (Fig. 1b). The richness and evenness of fecal microbiota measured by Faith’s PD index and Shannon index were significantly reduced in cirrhotic patients (both *p* < 0.001; Fig. 1c). Besides, the principal component analyses of unweighted UniFrac distance and Bray–Curtis distance showed a significant bacterial dissimilarity between these two groups (both *p* value = 0.001 by PERMANOVA test; Fig. 1d).

**Figure 2:**
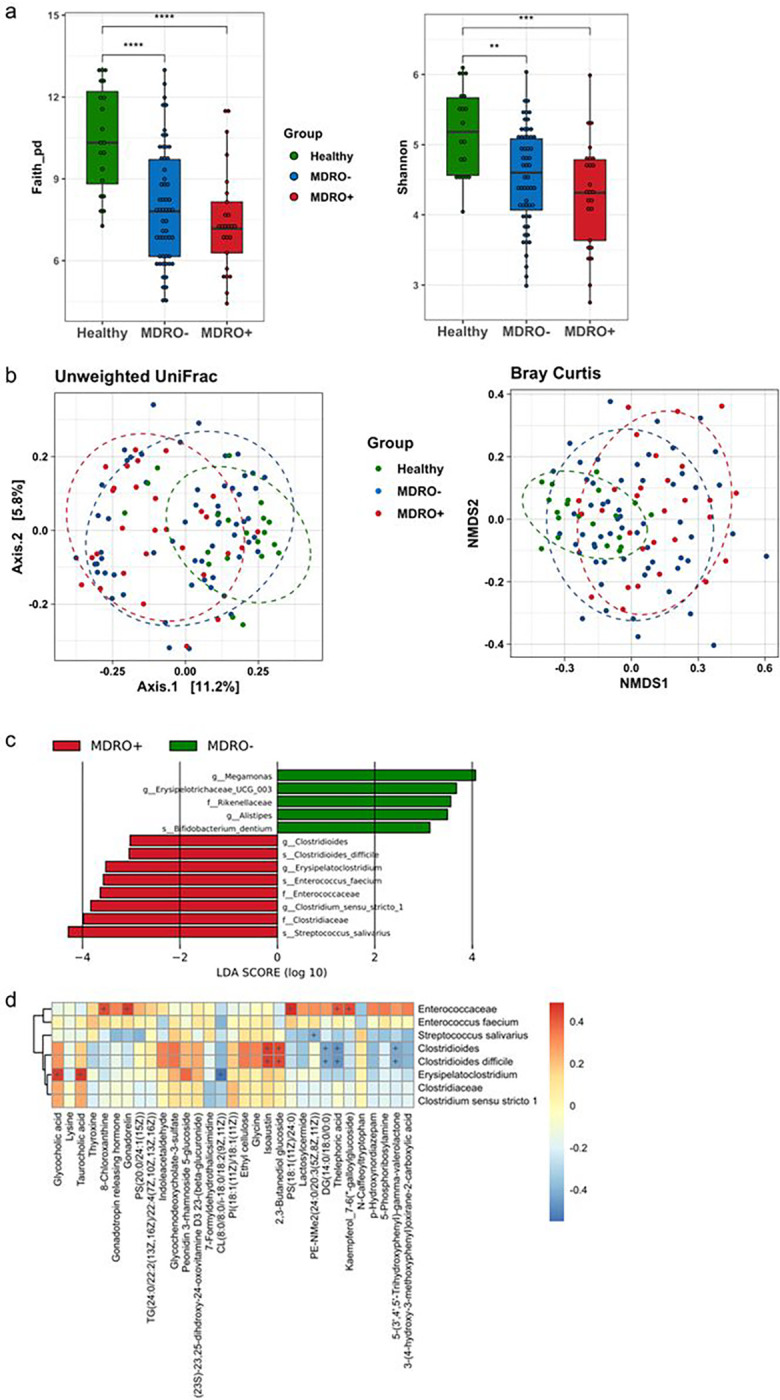
The composition and diversity of fecal microbiota among healthy subjects, cirrhotic patients with fecal MDRO carriage (MDRO+) and without MDRO carriage (MDRO−). (A) Alpha diversity indices of fecal bacteria measured by Faith’s PD index and Shannon index. (B) Principal coordinate analysis of fecal microbiota by unweighted Unifrac distance metrics and Bray–Curtis distance. (C) Linear discriminant analysis (LDA) effect size (LEfSe) showing differential abundance of taxa between fecal MDRO+ and MDRO− in cirrhotic patients. (D) Heatmap of the correlation analysis between the predominant bacterial taxa and plasma metabolites in cirrhotic patients with fecal MDRO carriage. MDRO, multidrug-resistant organism. ****p <0.0001, *** *p* <0.001, ** *p* <0.01

The phylogenetic diversity of fecal microbiota measured by Faith’s PD index and Shannon index decreased significantly in cirrhotic patients with and without MDRO carriage when compared with healthy controls (Fig. 2a). No statistical significance was observed between patients with cirrhosis with and without MDROs; however, a trend toward decreased alpha diversity in patients with cirrhosis with MDROs was observed. According to the unweighted UniFrac metrics, a significant dissimilarity of fecal microbiota was observed both between healthy controls and cirrhotic patients regardless of MDRO carriage (*p* = 0.001) and between cirrhotic patients with and without MDRO colonization (*p* = 0.033). However, the MDRO-associated microbial dissimilarity was not significant when measured using the Bray–Curtis distance (*p* = 0.134) (Fig. 2b). Furthermore, the results of LEfSe analysis showed a prominent abundance of *Streptococcus salivarius* in MDRO carriers, while Megamonas genus was abundant in MDRO non-carriers (Fig. 2c).

### Identification of Metabolomic Signature Associated with HE in Cirrhotic Patient With MDRO Carriage

**Figure 3:**
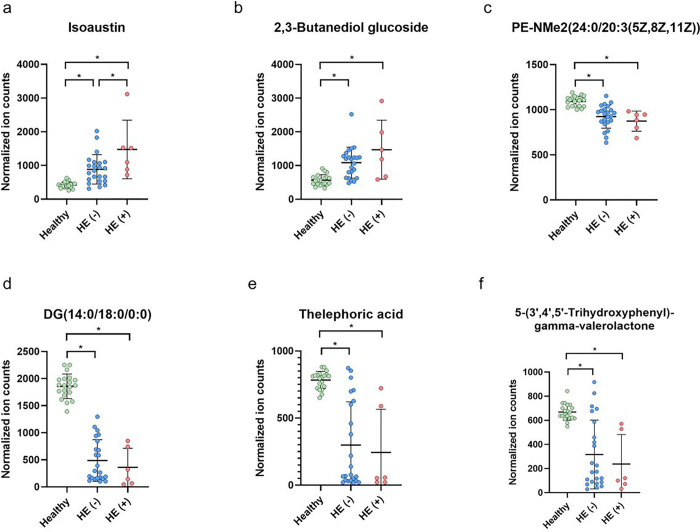
Expression levels of six metabolites associated fecal microbiota in healthy subjects and MDRO carriers with (HE+) and without hepatic encephalopathy (HE−). HE, hepatic encephalopathy.

Total 4869 untargeted metabolites in the plasma of cirrhotic patients by metabolomic analysis, of which 1618 metabolites were named. Thirty-two of the named metabolites that exhibited statistically significant differences in expression levels among healthy controls, cirrhotic patients with fecal MDROs, and those without MDROs were selected (**Suppl. Table 2**). The correlation analysis between the 32 metabolites and the dominant bacteria taxa in cirrhotic patients with MDRO carriage was performed (Fig. 2d). Six metabolites were identified to be correlated to specific microbiota expression patterns in patients carrying MDROs. A positive correlation between the presence of *Clostridioides difficile* and two metabolites—isoaustin and 2,3-butanediol glucoside—within cirrhotic patients carrying MDROs was observed (Fig. 2d). Notably, these 2 metabolites expressed significantly higher levels in MDRO carriers than in non-carriers (**Suppl. Table 2**). Conversely, a negative correlation was found between *Clostridioides difficile* and three other metabolites—DG (14:0/18:0/0:0), thelephoric acid, and 5-(3’,4’,5’-trihydroxyphenyl)-gamma-valerolactone—exhibiting diminished expression levels in MDRO carriers compared to non-carriers. In addition, *Streptococcus salivarius* exhibited a negative correlation with a single metabolite, PE-NMe2(24:0/20:3(5Z,8Z,11Z)), which was downregulated compared with MDRO non-carriers (**Suppl. Table 2**). There was no definitive positive correlation between *Streptococcus salivarius* and the broader spectrum of metabolites. The expression levels of these six metabolites in MDRO carriers who experienced HE and those who did not were further analyzed (Fig. 3); only isoaustin exhibited markedly higher expression in MDRO carriers who experienced HE than those who did not (Fig. 3a).

## Discussion

In this study, the cirrhotic patients with fecal MDRO colonization were at higher risk for occurrence of HE within 1 year compared to non-carriers. Furthermore, the presence of MDROs was associated with changes in gut microbiota diversity and alterations in specific metabolites, suggesting a connection between MDRO carriage and the increased risk of HE in cirrhotic patients.

High prevalence of MDRO colonization has been reported in cirrhotic patients with different conditions [7, 10, 24]. In cirrhotic patients waiting for liver transplant, the prevalence of MDRO colonization (from skin, oral and rectal samples) at listing was 20% and increased to 37% at transplantation [10]. Prado *et al*. observed that, among critically ill patients in the intensive care unit, patients with cirrhosis had a higher MDRO colonization rate than patients without cirrhosis at admission (28.7% vs 18.2%, respectively) [7]. The present study also showed a high fecal MDRO colonization rate for cirrhotic patients with general condition (33%), which was higher than healthy subjects (9.1%).

MDRO colonization, associated with subsequent MDRO infections, is an independent predictor for poor short-term survival for patients with cirrhosis [6, 7, 10, 24, 25]. Nevertheless, there was no difference in infection or mortality rates between MDRO carriers and non-carriers, and no MDRO infectious events occurred during the follow-up period in our patient population. Instead, we found a higher rate of overt HE in MDRO carriers. These differences may be attributed to variations in liver disease severity at enrollment, with previous studies mainly focusing on end-stage liver disease or critical conditions, often in hospitalized patients [6, 7, 10, 24]. In contrast, our study enrolled cirrhotic patients across all severities, with half being Child–Pugh class A, and a majority (80%) being outpatients. Therefore, the present study provided important information regarding MDRO carriage on cirrhosis-associated outcomes for both hospitalized and community-dwelling patients with cirrhosis.

In this study, higher serum LPS levels and fecal MDRO carriage helped to predict HE occurrence within the first year of follow-up. Patients with cirrhosis exhibit alteration of gut microbiota [17, 18, 26], which in turn increases the gut permeability and facilitates the translocation of bacterial products into systemic circulation, worsening endotoxemia. The altered microbiota compositions in cirrhotic patients might diminish the protective power of several *c*ommensals against colonization of pathogenic bacteria, including MDROs, in the host intestine [27]. LPS is a gut-derived endotoxin produced by Gram-negative bacteria that enters systemic circulation from the disrupted intestinal barrier in cirrhotic patients [28]. Therefore, patients with high LPS levels might have disturbed gut homeostasis and be more susceptible to colonization of pathogenic bacteria such as MDROs. Besides, a close correlation has been found between HE and the altered microbiota in cirrhosis [29–31]. Zhang *et al*. found a greater abundance of the gut ammonia-increasing bacteria *Streptococcus salivarius* in cirrhotic patients with minimal HE than in those without minimal HE [29]. Bajaj *et al*. reported that the colonic mucosal microbiota composition, rather than the stool microbiota, differed between patients with overt HE and without HE [30, 31], with a greater abundance of Enterococcus, Veillonella, Megasphaera, Bifidobacterium, and Burkholderia in patients with HE; in that study, the presence of Enterococcus, Megasphaera, and Burkholderia were linked to poor cognition and inflammation [30]. Reduced microbial diversity and change in microbiota composition have been reported in non-cirrhotic patients with MDRO carriage in comparison with those without MDRO carriage [11–13, 32]. Araos *et al*. showed that, in hospitalized non-cirrhotic patients, a greater abundance of Enterococcus spp. in MDRO carriers and microbiota belonging to Bacteroidales order in non-carriers, respectively [11]. Among older residents of nursing homes, *Odoribacter laneus*, and *Akkermansia muciniphila* were predominant in MDRO carriers, whereas *Blautia hydrogenotrophica* was predominant in non-carriers [32]. Consistently, in the present study, we observed that the gut microbiota composition was different between patients with and without MDRO carriage. We found a prominent abundance of *Streptococcus salivarius* in MDRO carriers, while Megamonas genus was abundant in MDRO non-carriers. Interestingly, the most abundant bacteria in MDRO carriers—*Streptococcus salivarius*— belongs to the urease-producing bacteria; this may partially explain why the rate of HE in this group was higher than that in the study by Zhang *et al* [29].

In addition to the gut microbiota, the bacterial associated metabolites can also influence the gut-liver-brain axis, which in turn contributes to HE development [33–36]. However, data regarding the interaction of MDROs and the gut bacteria associated metabolites with HE development in cirrhosis is limited. Our study demonstrated that of six of the metabolites correlated with the microbiota in MDRO carriers, a higher level of isoaustin was expressed in MDRO carriers with HE than in MDRO carriers without HE. However, the biological role of isoaustin in the human body has not yet been addressed. In our study, it remained unclear whether MDRO-associated metabolites are contributors or consequences of MDRO carriage. However, bacterial metabolism alterations could potentially contribute to antibiotic resistance by affecting energy production, modifying cell envelope compositions, and adjusting cell-to-cell interactions in biofilms [37, 38]. The role of metabolites in antibiotic resistance was supported by Ji’s study [39]. They found a suppression of glutathione oxidized and citrulline abundances in *Salmonella Derby* with drug resistance to third-generation cephalosporin; the susceptibility of multidrug-resistant *Salmonella Derby* to third-generation cephalosporin was further restored by exogenous glutathione oxidized or citrulline [39]. Taken together, changes of gut microbiota in cirrhosis might contribute to the metabolite alterations, with both factors promoting the growth of resistant bacteria in a given selective environment and inducing HE development. This suggests a dynamic relationship between the gut microbiota, metabolic changes, bacterial resistance, and HE development. Though the precise interaction between these factors was not disclosed in our study, our results provide new insights into the impact of MDRO carriage and metabolites in the cirrhotic population with occurrence of HE. Further research in this domain is crucial to unravel the mechanisms and potential therapeutic strategies to mitigate the impact of these interactions.

This study had some limitations. First, this was a single-center study performed in Taiwan. Therefore, the prevalence and distribution of MDROs might be different to those observed in other countries. Second, due to the observational nature of the study, detecting minimal HE events was challenging, preventing an assessment of the impact of microbiota and associated metabolites on patients with minimal HE. Third, many of the untargeted metabolites remained undiscovered, and only named metabolites were analyzed. Lastly, isoaustin was found to correlate with HE development in MDRO carriers; however, the biological role of isoaustin and its underlying mechanism remain unclear. The difficulty in obtaining these metabolites prevents us from conducting experimental studies to elucidate their roles. Nevertheless, our study uncovered a new impact on the correlation between MDROs, microbiota and the associated metabolites on HE.

In conclusion, the present study demonstrated the association between fecal colonization of MDROs, altered gut microbiota, metabolite modulation, and an elevated risk of HE occurrence among cirrhotic patients. The findings provide information to identify patients who may benefit from aggressive surveillance of fecal MDROs and to establish a treatment policy for decolonization in cirrhotic patients.

## Figures and Tables

**Table 1:** Antimicrobial resistance in fecal cultures between healthy subjects and cirrhotic patients

	Healthy (n=22)	Cirrhosis (n = 88)
MDRO colonized	2 (9.1)	29 (33.0)
Microorganism		
Gram negative		
*Escherichia coli* (ESBL)	1 (50)	17 (58.6)
*Klebsiella pneumoniae* (ESBL)	0 (0)	6 (20.7)
*Klebsiella pneumoniae* (CRE)	0 (0)	1 (3.4)
Gram positive		
*Enterococcus spp.* (VRE)	1 (50)	14 (48.3)
Methicillin-Resistant *Staphylococcus Aureus*	0 (0)	0 (0)
≥2 MDROs	0 (0)	6 (30.7)

The data are expressed as number (percent).

MDRO, multidrug-resistant organism; ESBL, extended spectrum β-lactamase; CRE, Carbapenem-resistant Enterobacteriaceae; VRE, vancomycin-resistant Enterococcus

**Table 2:** Baseline characteristics in cirrhotic patients with and without fecal carriage of MDROs

Variables	MDRO − (n = 59)	MDRO + (n = 29)	*p*-value
**Age, years**	61.75 (54.42–65.35)	56.36 (46.95–64.98)	0.067
**Male**	45 (76.3)	23 (79.3)	0.749
**Etiology of cirrhosis (%)**			
Viral/alcohol/others	42 (71.2)/ 12 (20.3)/ 5 (8.5)	15 (51.7)/ 8 (27.6)/ 6 (20.7)	0.142
**Laboratory**			
White blood cell (1000 /uL)	4.3 (3.2–5.3)	4.1 (3–5.45)	0.862
Platelet (1000/uL)	85 (58–120)	80 (46–117.5)	0.470
Sodium (mEq/L)	140 (138–142)	139 (136–141)	0.185
Creatinine (mg/dL)	0.84 (0.72–0.99)	0.80 (0.7–1)	0.535
Total bilirubin (mg/dL)	1.4 (1–2.2)	1.6 (0.9–3.7)	0.742
ALT (U/L)	27 (22–37)	32 (21.5–41)	0.260
Albumin (g/dL)	3.7 (3.3–4.1)	3.5 (3.1–3.95)	0.054
INR	1.26 (1.15–1.36)	1.34 (1.16–1.46)	0.131
LPS (ng/mL)	10.39 (8.73–15.1)	15.1 (9.99–20.91)	0.006
**Comorbidity**			
Diabetes mellitus	17 (28.8)	8 (28.4)	0.904
Hypertension	8 (13.6)	2 (6.9)	0.355
**Hepatocellular carcinoma**	10 (16.9)	5 (17.2)	0.973
**Presence of ascites**	31 (52.5)	14 (48.3)	0.707
**Presence of varices**	53 (89.8)	25 (86.2)	0.615
**Child-Pugh score**	6 (5–8)	7 (5–8)	0.177
**Child-Pugh class**			
A/B/C	35 (59.3)/ 19 (32.2)/ 5 (8.5)	13 (44.8)/12 (41.4)/4 (13.8)	0.417
**MELD score**	10.48 (8.98–13.43)	11.55 (8.37–16.44)	0.221
**Prior admission**			
**< 30 days**	8 (13.6)	10 (34.5)	**0.022**
**< 60 days**	15 (25.4)	11 (37.9)	0.247
**< 90 days**	19 (32.2)	13 (44.8)	0.227

The data are expressed as median (25th-75th percentiles), or number (percent).

MDRO, multidrug-resistant organism; ALT, alanine aminotransferase; INR, international normalized ratio; LPS, lipopolysaccharide; MELD, Model for End-stage Liver Disease

**Table 3:** Outcomes in cirrhotic patients with and without fecal carriage of MDROs within one year of follow-up

Variables	MDRO − (n = 59)	MDRO + (n = 29)	*p*-value
**SBP**	3 (5.1)	2 (6.9)	1.000
**Infections other than SBP**	6 (10.2)	4 (13.8)	0.615
**Hepatic encephalopathy**	2 (3.4)	6 (20.7)	**0.008**
**Variceal bleeding**	2 (3.4)	1 (3.4)	1.000
**Acute kidney injury**	13 (22.0)	5 (17.2)	0.600
**Newly onset ascites**	3 (5.1)	2 (6.9)	1.000
**Newly developed HCC**	4 (6.8)	2 (6.9)	1.000
**Mortality**	1 (1.7)	2 (6.9)	0.252
**Liver transplant**	1 (1.7)	0 (0)	1.000

The data are expressed as number (percent).

MDRO, multidrug-resistant organism; SBP, spontaneous bacterial peritonitis; HCC, hepatocellular carcinoma.

**Table 4:** Cox regression for hepatic encephalopathy within one year in cirrhotic patients

Variables	Univariate analysis			Multivariable analysis		
	HR	95%CI	p-value	HR	95%CI	p-value
**Age**	1.01	0.94–1.08	0.826			
**Male sex**	2.16	0.27–17.58	0.471			
**Type 2 diabetes mellitus**	0.34	0.04–2.78	0.316			
**Albumin (<3.5/ ≥ 3.5 g/dL)**	1.81	0.45–7.23	0.403			
**Sodium (<135 / ≥ 135mEq/L)**	3.99	0.80–19.82	**0.091**	4.60	0.65–32.60	0.127
**LPS (≥ 14.9/ < 14.9 ng/mL)**	14.97	1.84–121.83	**0.011**	11.01	1.24–97.50	**0.031**
**Child-Pugh class**						
A			Reference			
B	3.29	0.60–17.94	0.170	1.12	0.19–6.71	0.903
C	6.59	0.93–46.82	**0.060**	1.71	0.18–15.82	0.639
**MELD scores (≥ 10/ < 10)**	4.89	0.60–39.72	0.138			
**MDRO carriage**	6.68	1.35–33.13	**0.020**	5.47	1.02–29.31	**0.047**

HR, hazard ratio; CI, confidence interval; LPS, lipopolysaccharide; MDRO, multidrug-resistant organism

**Table 5:** Risk factors for fecal MDRO carriage

Variables	Univariate analysis			Multivariable analysis		
	OR	95%CI	p-value	OR	95%CI	p-value
**Age**	0.95	0.91–1.01	**0.077**	0.96	0.91–1.00	0.062
**Male sex**	1.19	0.41–3.51	0.749			
**Type 2 diabetes mellitus**	0.94	0.35–2.53	0.904			
**Albumin (< 3.5/ ≥ 3.5 g/dL)**	0.63	0.25–1.57	0.321			
**Sodium (<135 / ≥ 135mEq/L)**	1.25	0.28–5.62	0.775			
**LPS (≥ 11.9/ < 11.9 ng/ml)**	4.02	1.56–10.40	**0.004**	3.84	1.40–10.49	**0.009**
**Child-Pugh class**						
A			Reference			
B	1.70	0.65–4.46	0.280			
C	2.15	0.50–9.28	0.303			
**MELD scores (≥ 15/ < 15)**	2.30	0.84–6.29	0.106			
**Prior admission within 30 days**	3.36	1.15–9.77	**0.026**	1.04	0.38–2.85	0.945
**Presence of ascites**	0.84	0.35–2.05	0.707			

OR, odds ratio; CI, confidence interval; LPS, lipopolysaccharide; MDRO, multidrug-resistant organism

## Data Availability

Data available on request from the authors.
